# Evolution of Sangiovese Wines With Varied Tannin and Anthocyanin Ratios During Oxidative Aging

**DOI:** 10.3389/fchem.2018.00063

**Published:** 2018-03-15

**Authors:** Angelita Gambuti, Luigi Picariello, Alessandra Rinaldi, Luigi Moio

**Affiliations:** ^1^Division of Grape and Wine Sciences, Department of Agricultural Sciences, Viale Italia Angolo via Perrottelli, University of Naples Federico II, Avellino, Italy; ^2^Biolaffort, Bordeaux, France

**Keywords:** anthocyanins, tannins, red wine, oxidation, astringency, Sangiovese, aging

## Abstract

Changes in phenolic compounds, chromatic characteristics, acetaldehyde, and protein-reactive tannins associated with oxidative aging were studied in Sangiovese wines with varied tannin T/anthocyanin A ratios. For this purpose, three Sangiovese vineyards located in Tuscany were considered in the 2016 vintage. To obtain wines with different T/A ratios, two red wines were produced from each vinification batch: a free run juice with a lower T/A ratio and a marc pressed wine with a higher T/A ratio. An overall of six wines with T/A ratios ranging between 5 and 23 were produced. An oxidation treatment (four saturation cycles) was applied to each wine. Average and initial oxygen consumption rates (OCR) were positively correlated to VRF/mA (vanilline reactive flavans/monomeric anthocyanins) and T/A ratios while OCRs were negatively related to the wine content in monomeric and total anthocyanins. The higher the A content was, the greater the loss of total and free anthocyanins. A significant lower production of polymeric pigments was detected in all pressed wines with respect to the correspondant free run one. A gradual decrease of tannin reactivity toward saliva proteins after the application of oxygen saturation cycles was detected. The results obtained in this experiment indicate that VRF/mA and T/A ratios are among the fundamental parameters to evaluate before choosing the antioxidant protection to be used and the right oxidation level to apply for a longer shelf-life of red wine.

## Introduction

During red wine aging the exposure to low amounts of oxygen, as occurs during oak-barrel aging and/or micro-oxygenation, can produce several improvements. Among those most studied are: the increase of pigment stability (Atanasova et al., 2002; Cano-López et al., 2008), the decrease of astringency, and bitterness due to reactions of sensory active tannins (Cejudo-Bastante et al., 2011a; Gambuti et al., 2012), and the decrease of vegetative and reduction aroma (Cejudo-Bastante et al., 2011b; Gambuti et al., 2017). However, an excessive exposition to oxygen can also produce undesirable effects as the loss of red color and the appearance of oxidation taint. In recent decades the scientific community has widely investigated phenomena occurring during wine aging and the main chemical reactions involved are known (du Toit et al., 2006; Waterhouse and Laurie, 2006). Today finding simple useful models able to predict how the wine is going to respond to oxygen exposure is one of the most challenging goals. These models and knowledge of the wine's key compounds affecting wine evolution in contact with oxygen are fundamental for a proper management of wine aging in both the winery and on the market. In this context, studies of the parameters affecting oxygen consumption rates (OCR) are a landmark. Tannin chemical structure (Vivas and Glories, 1996; Ferreira et al., 2015), native anthocyanin composition (Carrascon et al., 2015), copper and iron concentrations (Danilewicz, 2007), pH (Singleton, 1987), and SO_2_ (Danilewicz, 2007; Danilewicz et al., 2008; Gambuti et al., 2015) are among the wine parameters best correlated to OCR. A great amount of information obtained in recent decades has led to improvements in the shelf-life management of red wines. However, given the complex nature of wine and the fact that most of these studies deal with individual compounds and/or simplified model systems, more experiments on real wines are necessary. Among the vast group of wine phenolics, anthocyanins and tannins, together with wine color and nonbleachable (SO_2_-resistant) pigments, have been reported as crucial for the quality of red wine, and have turned out to be well correlated with its commercial value (Mercurio et al., 2010; Kassara and Kennedy, 2011). Given this, several authors investigated parameters affecting wine color development and production of stable pigments. Ristic et al. (2010) showed that wine tannins and anthocyanin concentration are the strongest predictors of stable wine color development. Recently Merrell et al. (2017) observed that the higher the anthocyanin and tannin concentrations extracted during fermentation were, the higher the wine color density and content of total SO_2_-resistant pigments. Concerning color stability Singleton and Trousdale (1992) observed instead that increasing anthocyanin and tannin concentrations does not always mean an increase in SO_2_ resistant pigment. This finding has suggested that an ideal tannin to anthocyanin ratio (T/A) exists. Other authors subsequently highlighted the importance of T/A for polymeric pigments formation (Brossaud et al., 1999; Fulcrand et al., 2004). It is therefore likely that this ratio determines wine evolution under low oxygen exposure. However, due to the difficulty of manipulating this ratio while keeping other winemaking factors constant, the relationship between oxygen consumption rate and tannin/anthocyanin ratio has never been evaluated for wines produced from the same grape lot. Moreover, the evaluation of the effect of this ratio on the evolution of sensory active tannins during wine oxygen exposure has never been reported.

In this study wines with different T/A ratios were obtained from the same grape (Sangiovese Vitis vinifera L.) applying two levels of marc pressing after maceration. Sangiovese grape variety was chosen because is the most widely planted vine variety in Italy (OIV, 2017)^1^. The initial and average OCR after four saturation cycles as well as changes in phenolic compounds, chromatic characteristics, and sensory active tannins were determined. The evolution of perceptible tannins was estimated by considering the ability of these molecules to precipitate salivary proteins (Gambuti et al., 2006) and BSA (Harbertson et al., 2003) simulating the astringency perception.

## Materials and methods

### Wines

Six wines were produced in the 2016 vintage. Wines were obtained from grapes belonging to three wineries located in the Chianti DOCG area and because of their richness in anthocyanins were classified as HA (wines with a High Anthocyanin level), MA (wines with a Medium Anthocyanin level) and LA (wines with a Low anthocyanins level). From each vineyard two wines were produced as follows: grapes were destemmed and crushed; the must was treated with K_2_S_2_O_5_ (40 mg/kg of grapes); musts were inoculated with F83 yeast at 20 g/hL (Laffort, Bordeaux, France); fermentation took place at 25°C and the maceration of the pomace lasted 12 days; 50 g/hL of a mixture of ammonium salts and thiamine were added during maceration; the cap was immersed twice a day for the first 2 days, 4 times from the third to the seventh day and 1 time the remaining 4 days of maceration. Subsequently, the must was pressed at 0.2 bar to obtain the free run juice (f) and at 8 bar to obtain the pressed run juice (p). A total of 6 wines were obtained and classified as: HAf (High Anthocyanins level from free run juice), HAp (High Anthocyanins level from pressed run juice), MAf (Medium Anthocyanins level from free run juice), MAp (Medium Anthocyanins level from pressed run juice), LAf (Low Anthocyanins level from free run juice), LAp (Low Anthocyanins level from pressed run juice). All wines were inoculated with lactic bacteria (LF16 direct, Laffort, Bordeaux, France) at 1 g/hL. Sulfur dioxide was then added to the wine. Base parameters of wines at the end of maceration are showed in Table 1.

**Table 1:** Initial wine composition and initial and average oxygen consumption rates of Sangiovese wines.

	**HAf**	**HAp**	**MAf**	**MAp**	**LAf**	**LAp**
EtOH (%)	14.79 ± 0.25	14.75 ± 0.09	14.35 ± 0.16	14.09 ± 0.28	12.32 ± 0.35	12.32 ± 0.05
pH	3.28 ± 0.02	3.36 ± 0.03	3.35 ± 0.05	3.40 ± 0.07	3.45 ± 0.02	3.46 ± 0.00
free SO_2_ (mg/L)	22.72 ± 0.5	21.44 ± 0.5	23.68 ± 3.6	22.72 ± 0.5	22.54 ± 0.2	22.72 ± 0.5
total SO_2_ (mg/L)	41.6 ± 0.9	45.44 ± 0.9	50.88 ± 0.5	44.16 ± 2.7	41.04 ± 1.9	43.48 ± 1.8
total acetaldehyde (mg/L)	19.14 ± 0.30	18.57 ± 0.33	11.48 ± 0.43	12.78 ± 0.06	16.41 ± 0.24	26.55 ± 0.27
iron (mg/L)	0.438 ± 0.023	0.228 ± 0.009	0.246 ± 0.011	0.065 ± 0.003	0.935 ± 0.038	0.279 ± 0.021
copper (mg/L)	0.208 ± 0.008	0.296 ± 0.011	0.095 ± 0.004	0.167 ± 0.005	0.017 ± 0.001	0.246 ± 0.021
VRF/mon anth	10.6	11.2	9.1	10.2	30.5	38.9
T/A^*^	5.9	8.6	4.9	6.2	13.3	22.7
initial OCR (mg/L/day)	3.620 ± 0.007	3.775 ± 0.503	3.545 ± 0.282	3.598 ± 0.798	4.608 ± 0.49	3.790 ± 0.003
average OCR (mg/L/day)	1.101 ± 0.014	1.296 ± 0.01	1.402 ± 0.048	1.355 ± 0.084	2.876 ± 1.031	3.302 ± 0.022

*HAf, High Anthocyanins level from free run juice; Hap, High Anthocyanins level from pressed run juice; MAf, Medium Anthocyanins level from free run juice; Map, Medium Anthocyanins level from pressed run juice; LAf, Low Anthocyanins level from free run juice; Lap, Low Anthocyanins level from pressed run juice*.

* *T/A, BSA-reactive tannins (mg/L)/total anthocyanins (mg/L)*.

### Oxidation process

The oxidation experiment consisted of four consecutive air saturation cycles. The chemical composition of the wines before and after oxidation was extensively characterized. Two 1 L bottles of each wine containing PSt3 oxygen sensors (Nomacorc SA, Thimister-Clermont, Belgium) were saturated with air by adding a gentle flow of oxygen through a mini-compressor for 15 min until the oxygen level of the wine reached 6.6 mg/L. At the end of each saturation, the bottles containing the saturated wines were filled up completely and were carefully closed under nitrogen avoiding any headspace. Wines were stored in an incubator in the dark at 25°C and dissolved oxygen level was monitored at least once a day with a Nomasense oxygen analyzer from Nomacorc S.A. (Thimister-Clermont, Belgium). The oxidation cycle was considered finished once O_2_ levels dropped to 10% of the initial concentration or after a week. Then the bottles were opened and a small sample for intermediate analysis was taken. The remaining wine was taken out for a new saturation cycle. Data of oxygen concentration were transformed into oxygen consumption, simply by subtracting the measured O_2_ level at each data point from the measured O_2_ level at the beginning of the corresponding saturation. The accumulated oxygen consumption was then plotted vs. time, and the initial and average oxygen consumption rate of that wine for saturations 2–4 was determined as previously reported by Ferreira et al. (2015). All saturation treatments were made in duplicate.

### Spectrophotometric analyses

Color intensity (Abs 420 nm + Abs 520 nm + Abs 620 nm) and hue (Abs 420 nm/Abs 520 nm) were determined by spectrophotometric measuring using a Shimadzu UV-1800 (Kyoto, Japan) UV spectrophotometer. All analyses were carried out in duplicate. The CIELAB parameters (L^*^, a^*^, b^*^) were determined by using the software Panorama (PANORAMA SOFTWARE UPGRADE PATH), following the recommendations of the Commission Internationale de L'Eclariage (CIE). Color differences (ΔE/ab) were calculated as the Euclidean distance between two points in the 3D space defined by L^*^, a^*^, and b^*^.Total anthocyanins, bovine serum albumin (Sigma Aldrich SRL, Milano, Italy) reactive tannins (BSA reactive tannins), short polymeric pigments (SPP), and large polymeric pigments (LPP) were determined by the Harbertson–Adams assay (Harbertson et al., 2003). Vanillin reactive flavans (VRF) were determined as reported in Gambuti et al. (2015). All analyses were conducted through two experimental replicas and two analytical replicas.

### High-performance liquid chromatography analyses of acetaldehyde

Acetaldehyde was determined by HPLC after derivatization reaction with 2,4-dinitrophenylhydrazine reagent (Aldrich chemistry) as reported by Han et al. (2015). Briefly, wine sample aliquots (100 μL) were dispensed to a vial, followed by the addition of 20 μL of freshly prepared 1,120 mg/L SO_2_ solution, 20 μL of 25% sulfuric acid (Carlo Erba reagent 96%), and 140 μL of 2 g/L 2,4-dinitrophenylhydrazine reagent. After mixing, the solution was allowed to react for 15 min at 65°C and then promptly cooled to room temperature. Carbonyl hydrazones were analyzed by HPLC using a HPLC Shimadzu LC10 ADVP apparatus (Shimadzu Italy, Milan), consisting of a SCL-10AVP system controller, two LC-10ADVP pumps, a SPD-M 10 AVP detector, and an injection system full Rheodyne model 7725 (Rheodyne, Cotati, CA) equipped with a 50 μL loop. The separation was carried out on a Waters Spherisorb column (250 x 4.6 mm, 4 μm particles diameter) equipped with a guard column. Optimum efficiency of separation was obtained using a flow rate of 0.75 mL/min, column temperature of 35°C; mobile phase solvents were: (A) 0.5% formic acid(Sigma Aldrich ≥ 95%) in water milli-Q (Sigma Aldrich) and (B) acetonitrile (Sigma Aldrich ≥ 99,9%); gradient elution protocol was: 35% B to 60% B (*t* = 8 min), 60% B to 90% B (*t* = 13 min), 90% B to 95% B (*t* = 15 min, 2-min hold), 95% B to 35% B (*t* = 17 min, 4-min hold), total run time, 21 min. Eluted peaks were compared with derivatized acetaldehyde standard. All analyses were conducted through two experimental replicas and two analytical replicas.

### High-performance liquid chromatography analyses of anthocyanins

The separation of the monomeric anthocyanins was carried out as previously reported (OIV, 2009) in the HPLC system previously described (par 2.3) in conjunction with a column heating device set at 40°C, with a C18 column, Waters Spherisorb column (250 × 4.6 mm, 4 μm particles diameter) with pre-column. All the samples were filtered through 0.45 mm, Durapore membrane filters (Millipore—Ireland) into glass vials and immediately injected into the HPLC system. A 50 μL loop was used. Briefly, elution was carried out by using a flow rate of 0.80 mL/min. Eluents were: solvent A consisting of water milli-Q (Sigma Aldrich)/formic acid(Sigma Aldrich ≥ 95%)/acetonitrile(Sigma Aldrich ≥ 99.9%) (87:10:3) v/v and, solvent B consisting of water/formic acid/acetonitrile (40:10:50) v/v. The following gradient was used: zero-time conditions 94% A and 6% B, after 15 min the pumps were adjusted to 70% A and 30% B, at 30 min to 50% A and 50% B, at 35 min to 40% A, and 60% B, at 41 min, end of analysis, to 94% A and 6% B. After 10-min equilibrium period the next sample was injected. For calibration the external standard method was used: the calibration curve was plotted for the malvidin-3-monoglucoside (Extrasynthese, Lyon, France) on the basis of peak area. The concentration of the following monomeric anthocyanins was determined: delphinidin 3-glucoside, cyanidin 3-glucoside, peonidin 3-monoglucoside, malvidin 3-glucoside, vitisin B, malvidin 3-(6^II^-acetyl)-glucoside. The concentration was expressed as mg/L of malvidin-3-monoglucoside. All analyses were conducted through two experimental replicas and two analytical replicas.

### The saliva precipitation index (SPI)

Tannins reactive toward saliva proteins were measured by the Saliva Precipitation Index (SPI) (Rinaldi et al., 2012). Briefly, wine tannins reacted with human saliva at 37°C (in-mouth temperature) for 5 min. After cold precipitation (4°C), wine tannins bound to proteins were separated from supernatant, which was analyzed by chip electrophoresis. The commercial Experion Pro260 analysis kit and the Experion system were used for the SPI determination. The saliva samples (before and after the binding reaction) were treated with the Experion reagents in accordance with the instructions, and then separated on the Experion automated electrophoresis station according to the manual instruction. The SPI was calculated by the percentage reduction of the fluorescence signal of salivary proteins compared to control saliva (before reaction) (Rinaldi et al., 2014). Results are expressed as mg/L of gallic acid equivalent (GAE).

### Metal analyses

Total iron and total copper analyses were determined as reported in multielemental analysis using ICP-MS OIV-MA-AS323-07 (OIV, 2009) by the Laboratorio Ambientale Gamma (Avellino, Italy).

### Statistical analysis

Fisher's least significant differences (LSD) procedure was used to discriminate among the means of the variables when the variables fulfilled the parametric conditions for chemical and OCR data. When the variances were not homogeneous, data were analyzed using Kruskall–Wallis test and significant differences were established by using Notched Box Plots. Differences of *p* < 0.05 were considered significant. Partial least square (PLS) regression was carried out using the PLS module of the XLSTAT software (Addinsoft, 2009)^2^ to predict OCR from the set of data collected.

## Results

### Wine oxygen consumption in air saturation cycles and initial wine composition

**Figure 1:**
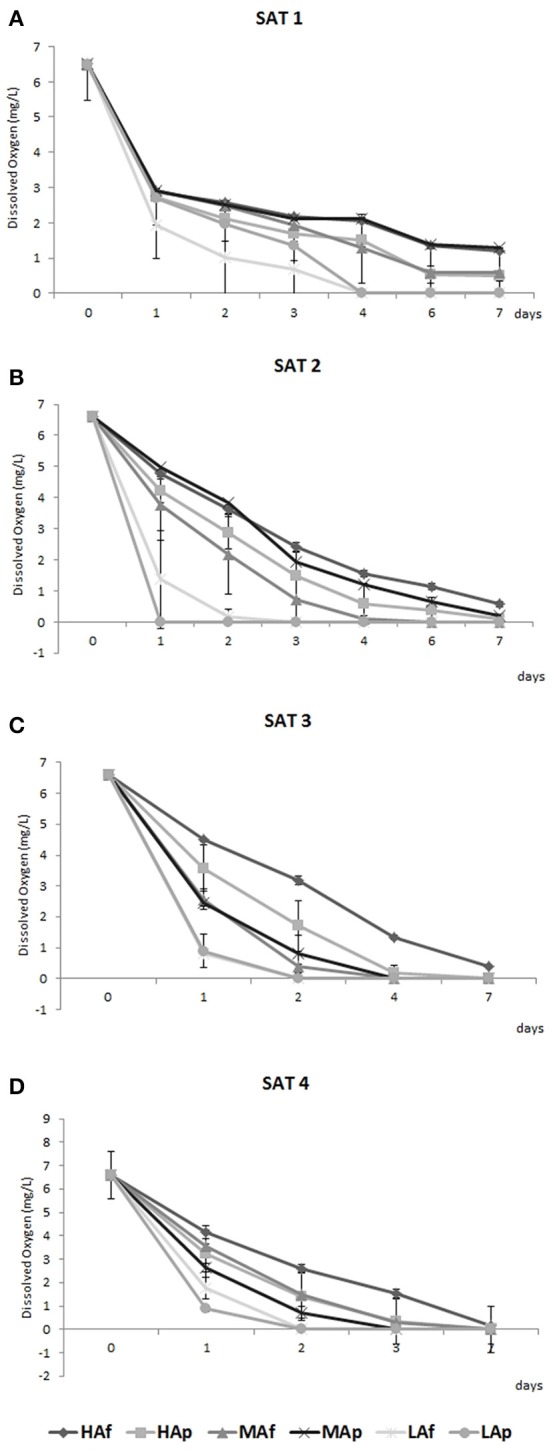
Average oxygen concentrations (mg/L) measured in each wine sample after the first **(A)** (SAT1), the second **(B)** (SAT2), the third **(C)** (SAT3), and the fourth **(D)** (SAT4) saturation.

Wines distributed in independent bottles containing oxygen sensors were oxidized along four consecutive air saturation cycles in duplicate. Figure 1 shows dissolved oxygen contents for each saturation cycle. As already observed (Ferreira et al., 2015) the kinetics of oxygen consumption in red wines are complex and several differences between the initial consumption rate and the overall trend during the saturation cycles can be observed. Initial and average oxygen consumption rates were calculated as described by Ferreira et al. (2015). In agreement with previous studies (Ferreira et al., 2015; Carrascón et al., 2017) oxygen consumption rates at the beginning of the first saturation cycle are the highest in the cycles (initial OCR in Table 1). However this pattern was not repeated in the same way for all wines: it was confirmed in LA wines in all saturation cycles and less evident in HA wines in SAT2-SAT4 saturation cycles. Values of initial and average OCR are in the range previously detected for Garnacha and Tempranillo red wines (Carrascón et al., 2018) and consistent with those reported elsewhere for some white and rosè wines (Carrascón et al., 2017). Average OCR values of wines increased passing from HA to LA wines while no effect of marc pressing was detected. With the aim to investigate the reason for such differences and the contribution of wine initial composition to initial and average OCRs, a correlation analysis was performed (Table 1). The average OCR, and to a lesser extent initial OCR, are positively correlated to VRF/mA and T/A ratios and acetaldehyde (Table 2). Initial and average consumption rates are instead negatively related to the wine content in monomeric and total anthocyanins; for all these analytes the negative correlation is stronger with average OCR (Table 2). Similar correlation for pigments, monomeric anthocyanins and acetaldehyde were previously reported (Ferreira et al., 2015; Carrascón et al., 2017).

**Table 2:** Significant correlation coefficients between initial and average oxygen consumption rates and the chemical composition of the Sangiovese wines before the oxidation (*p* < 0.05).

	**Initial rates**	**Average rates**
monomeric anthocyanins (mg/L)	−0.660	−0.964
total anthocyanins (mg/L)	−0.560	−0.950
LPP+SPP	−0.289	ns
VRF (mg/L)	0.194	−0.207
BSA reactive tannins (mg/L)	−0.146	−0.469
SPI (g/L)	ns	ns
abs 420	−0.333	−0.732
abs 520	−0.366	−0.748
abs 620	−0.219	−0.546
CI	−0.337	−0.721
hue	ns	ns
VRF/mon. anth.	0.592	0.988
T/A^*^	0.392	0.926
iron (mg/L)	ns	ns
copper (mg/L)	ns	ns
EtOH	−0.689	ns
pH	ns	ns
free SO_2_ (mg/L)	ns	ns
tot SO_2_ (mg/L)	ns	ns
acetaldehyde (mg/L)	0.113	0.598

* *T/A, BSA-reactive tannins (mg/L)/total anthocyanins (mg/L); ns, not significant*.

A PLS model explaining average oxygen consumption rates could be built:

Average OCR = 1.32195-0.00075^*^mon. anth. −0.00039^*^tot. anth. +0.00127^*^VRF −0.00034^*^BSA reactive tannins −0.11301^*^abs420 −0.00721^*^abs520 −0.20000^*^abs620 −0.02026^*^Ci + 0.03014^*^VRF/mA + 0.01617^*^T/A + 0.00256^*^acetaldehyde (mg/L).

The Q^2^ cumulated index, which measures the global goodness of fit and the predictive quality of the model, is 0.955 for the first two components and 0.999 for the first three. The model with 2 PCs explained 97.43% of the original variance, and indicated that average consumption rates are positively related to the wine content in VRF/A and T/A ratios, VRF and acetaldehyde, and negatively correlated to monomeric and total anthocyanins, BSA reactive tannins, color intensity and 420, 520, and 620 nm absorbances.

### Variation of pigments and chromatic characteristics

**Figure 2:**
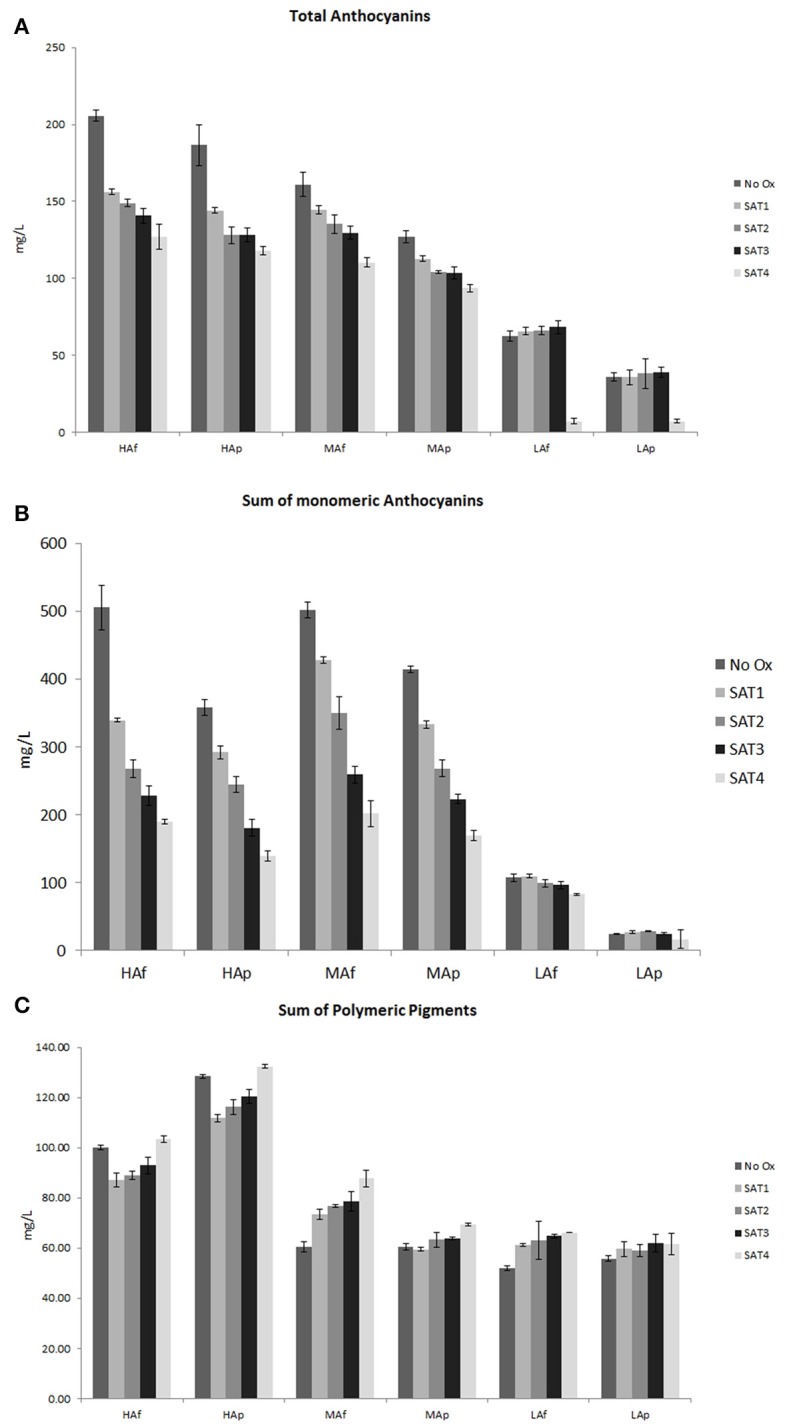
Total **(A)**, sum of monomeric **(B)** and polymeric **(C)** pigments (mg/L of malvidin−3-monoglucoside) of wines before and after each saturation cycle.

After oxygen saturation a loss of monomeric and total anthocyanins and a concomitant production of new pigments was detected for all wines (Figure 2). After the fourth saturation cycle, the greatest loss of native anthocyanins was observed in LA wines while a significant lower production of polymeric pigments was detected in all pressed wines with respect to free run juice ones (Figure 2). As expected, changes in pigment composition strongly affected chromatic characteristics of wines after oxygen saturations (Tables 1, 2 in Supplementary Material). The values of the different color coordinates measured during saturation cycles are showed in Table 2. Lightness L^*^ increased in HAf, HAp, MAf, and LAp through the saturation cycles indicating that at the end of the study the samples are less dark. In wine the progressive loss of pigments observed during aging would be responsible for the increase in lightness (Rivas et al., 2006; Heras-Roger et al., 2014). The a^*^ coordinate decreased for HA and MA wines (from 6 to 36% of initial values) which indicated a statistically significant reduction of the red color. This color drop can be due to the reduction of these pigment concentrations but also to the involvement of these pigments in the formation of new pigments with different spectral properties and to the reduction of the copigmentation effect due to the loss of copigments. A significant increase of yellow color measured by the b^*^ coordinate was instead observed for LA wines. This increase, previously observed during wine aging, is due to the formation of orange–yellow pigments as orange pigments resulting from oxidation of compounds such as flavanols or from reactions of anthocyanin pigments (Pérez-Magariño and González-San José, 2004). In all wines except for LAf, the C^*^ parameter decreased, reflecting a color intensity loss in the oxygen saturated wines. An important parameter to understand the possible effect of a treatment on human visual perception is ΔE. It was calculated considering the color coordinates of the wines before and after all oxygen saturation cycles (Table 1 in Supplementary Material). According to Mokrzycki and Tatol (2012) a subsequent ΔE classification can be considered to understand if two samples show a visible difference: 0–0.5 (not noticeable), 0.5–1.5 (slightly noticeable), 1.5–3.0 (noticeable), 3.0–6.0 (well visible), and >6.0 (great). In all six experimental wines the ΔE values calculated are superior to 1 meaning that a standard observer can see a difference in color. Clear visible differences can be observed for HA wines already after the first saturation and they slightly increase in further saturations. Even for MA and LA wines 2.51 < ΔE < 0.99 a difference in color can be noticed. The strong effect of oxygen saturation on HA wines is evident also considering the most usual parameter CI (Table 2 in Supplementary Material). It showed a significant decline (close to 20%) already after the first saturation cycle, a slight increase was instead observed in MAf (7.7%) and a slight decrease in LAp (7%). In the case of HA wines, it is likely that most of the loss of CI after the first saturation is due to the lower value of native anthocyanins but also to the lower contribution to red wine color of copigmentated anthocyanins. For all HA and MA wines a concomitant slight increase (<7%) of hue was detected.

### Variation of protein reactive tannins and vanilline reactive flavans

**Figure 3:**
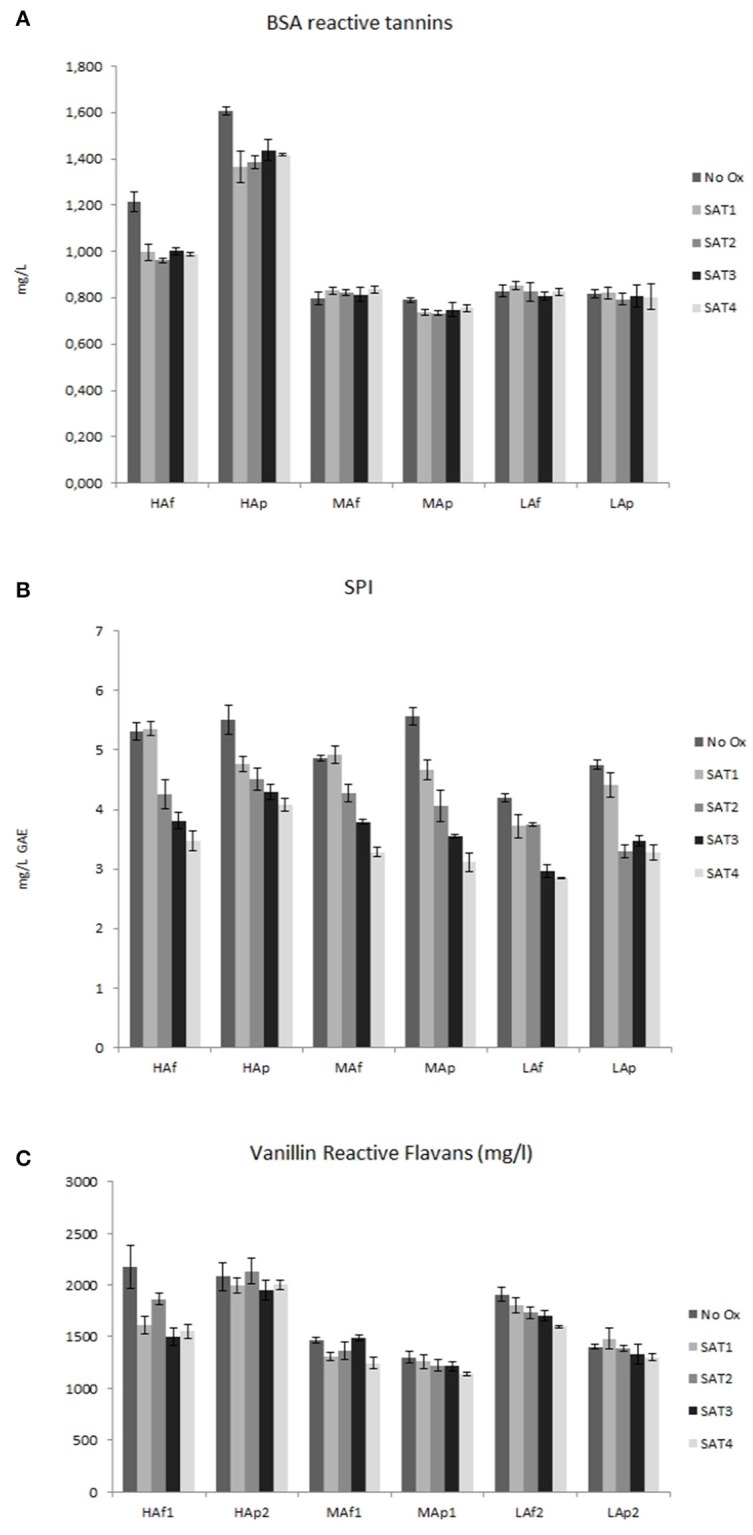
BSA reactive tannins (mg/L of catechin) **(A)**, SPI (mg/L of gallic acid equivalent GAE) **(B)**, and Vanilline Reactive Flavans (VRF) (mg/L of catechin) **(C)** of wines before and after each saturation cycle.

The amounts of tannins precipitable by saliva proteins (SPI) and BSA are showed in Figure 3. The gradual decrease of SPI detected during saturation cycles indicates a potential decrease of astringency of these wines after reaction with oxygen. The formation of higher molecular weight structures and/or in changes of composition and flexibility of molecules during oxidative stress are processes likely involved (Vallverdú-Queralt et al., 2017). VRF values can give a possible confirmation of this hypothesis owing to the previous findings that a decrease of the value of VRF is an indirect indication of an increase of polymerization degree of condensed tannins (Gambuti et al., 2012; Picariello et al., 2017). After 4 saturation cycles values of these parameters decreased for all samples (from 4 to 29%). As already observed for polymeric pigments, so too for VRF the loss is higher in free run juice wines compared to pressed ones, likely due to the presence of higher content of anthocyanins reacting with monomeric and condensed flavanols. These results are in partial contrast with findings that BSA reactive tannins decreased only for HA wines. However, not all condensed tannins were considered in this assay and not all those reacting with saliva. It is known that the efficacy of condensed tannins for BSA precipitation increases with increasing degree of polymerisation (or size) from trimers to octamers (Harbertson et al., 2014). Previous works also showed that during oxidation of red wines SPI followed a trend different from BSA reactive tannins (Picariello et al., 2017).

## Discussion

### Wine oxygen consumption in air saturation cycles and initial wine composition

The kinetics of oxygen consumption in red wines are complex (Ferreira et al., 2015) and, as expected, several differences between the initial consumption rate and the overall trend during the saturation cycles can be observed. These results confirm the existence of different oxidation mechanisms and indicate that the newly formed oxidized compounds are less prone to consume oxygen during subsequent saturation cycles. Furthermore it is likely that the new larger polymeric oxidizable derivatives are different from wine to wine (Boulton et al., 1996). The presence of different amounts of transition metals and tartaric and malic acids interfering with oxidation mechanisms can also account for differences among wines (du Toit et al., 2006; Danilewicz, 2014).

Thanks to the PLS regression, a positive correlation between the average consumption rates and VRF/mA and T/A ratio was detected for the first time (Table 2). The influence of T/A ratios during an oxidative process on polymerization reactions had already been observed (Gambuti et al., 2017; Picariello et al., 2017), yet until now no information on the kinetics of oxidation had previously been reported. The relationship between OCR and studied ratios is explainable considering that ortho-hydroxyl groups on the B ring of flavanols (VRF and T) promote the oxidation to quinone. As this hydroxylation pattern is lacking in malvidin, the most aboundant wine anthocyanins (A), the formation of quinones should be instead slower (Boulton et al., 1996). Furthermore the reaction of malvidin with the main product of oxidation, the acetaldehyde, is also slower than for catechin (Sheridan and Elias, 2016). The key role of anthocyanins is consistent with previous works (Carrascon et al., 2015; Carrascón et al., 2017).

Concerning acetaldehyde our results contrast with Carrascón et al. (2018) who found a negative correlation with OCR as consequence of an indirect effect of the presence of acetaldehyde-reactive polyphenols (ARPs). Because the reaction of some of these ARPs (e.g., catechin) with acetaldehyde is fast (Sheridan and Elias, 2016), wines with higher amounts of acetaldehyde should be wines with smaller amounts of ARPs. However wine is a complex mixture containing multiple ARPs and other acetaldehyde reactants (Peterson and Waterhouse, 2016) (among them the highly reactive SO_2_) and it is presently not possible to make an easy and simple correlation with a specific group of compounds.

Albeit the importance of metals in allowing polyphenol oxidation (Danilewicz, 2007) is crucial, no significant correlation between the OCRs and total iron and copper content was detected. This result is probably due to the fact that the metal speciation was not determined (Danilewicz, 2013; Ferreira et al., 2015). In agreement with Rousseva et al. (2016) the total copper correlates more closely with oxygen consumption in the wine compared to total iron but, the residual and cationic forms of copper provide the largest contribution. As for iron, Danilewicz (2016) observed that Fe(III) oxidation of polyphenols is somewhat slower than the reaction of Fe(II) with oxygen, which is instead greatly accelerated by Cu. In addition Kontoudakis et al. (2017) observed a strong dependence on iron concentration in the increase in the oxidation state of Fe (Fe(III) vs. Fe(II)). However, in our samples concentration of total iron are well below values detected in some Chardonnay wines by Kontoudakis et al. (2017). About metal speciation, unfortunately only the total metal contents was determined in our experiment.

### Variation of pigments and chromatic characteristics

Different mechanisms can explain the loss of anthocyanins detected after each saturation cycle. First, acetaldehyde deriving from the oxidation of ethanol gives numerous reactions with free native anthocyanins producing pyranoanthocyanidins and new polymeric pigments (Fulcrand et al., 2004). Second, oxygen addition can activate the reactions between free anthocyanins and flavan-3-ols leading to more stable pigments (Escribano-Bailón et al., 2001). Finally, the fast consumption of native anthocyanidins in reactions with quinones produced in the first stages of oxidation from vicinal diphenols (Sarni-Manchado et al., 1997). The evidence of a lower negative correlation with initial OCR compared to average OCR support this last hypothesis. In all wines an increase of polymeric pigments (10–45%) was detected at the end of the fourth saturation cycle consistent with other authors applying oxygen saturations (Carrascon et al., 2015) and micro-oxygenation (Cano-López et al., 2010; Gambuti et al., 2015). However different trends among wines are observed. It is clear that the loss of native pigments as well as the formation of polymeric pigments is strongly related to the wine's original content of anthocyanins and compounds reacting with anthocyanins. After the oxidation of HAp, Map, and LAp wines a lower formation of polymeric pigments was observed indicating that the effect of T/A ratio is significant. Previous studies even showed that the T/A ratio affected the production of polymeric pigments during bottle aging (Gambuti et al., 2017) and after a strong oxidation of red wine (Picariello et al., 2017). The composition of grape native tannins (Bindon et al., 2014) and the level of marc pressing and related release-diffusion of phenolics to medium (Gambuti et al., 2004; Cerpa-Calderón and Kennedy, 2008; Setford et al., 2017) are others crucial factors that significantly influence the formation of polymeric pigments in wines.

Transformation of colorant matter with oxygen saturation cycles determine a significant change in chromatic characteristics (loss of CI and increase of hue). Differences among ΔE calculated before and after the four saturation cycles suggests that the higher the content of native and total anthocyanins of a wine is, the greater the effect of oxygen on chromatic characteristics. A negative effect of oxygen saturation on CI was not observed by Carrascon et al. (2015) after five saturation cycles, while authors detected a comparable increase of Abs 420 nm/Abs 520 nm ratio. A significant loss of CI was instead detected by Cejudo-Bastante et al. (2011b) after a micro-oxygenation treatment on Cencibel red wines and by Picariello et al. (2017) after the addition of hydrogen peroxide on a Lambrusco wine with added condensed tannins. As the red wines treated by Carrascon et al. (2015) were more aged than our samples, it is possible that the initial lower content of monomeric (more reactive) anthocyanins can justify the different trends detected.

### Variation of protein reactive tannins and vanilline reactive flavans

One of the reasons why some red wines benefit from low oxygen exposure occurring during barrel/bottle aging and/or micro-oxygenation is the consequent loss of astringency. The most established mechanism for astringency involves the interaction between wine tannins and saliva proteins (Ma et al., 2014). As wine tannins are a heterogeneous mixture containing a range of different polymer sizes, subunit compositions, and subunit linkages and as a complete analysis of all phenolic composition is difficult to realize, the analysis of protein precipitation after the reaction with wine is an indirect simple measure of potential astringency. The gradual decrease observed for SPI and VRF through the saturation cycles indicates a polymerization and consequent lower reactivity of tannins. This partially contrasts with results on BSA reactive tannins that decreased only for HA wines. However differences in reactivity toward BSA and saliva are not ruled out. Bovine serum albumin (BSA) is a single polypeptide chain used as standard globular protein to predict grape and wine astringency (Harbertson et al., 2003). Harbertson et al. (2014) showed that monomers and dimers of flavan-3ols did not precipitate BSA while up to 93% of octamer was precipitated. Saliva is instead a much more complex mixture of proteins and, among them the great family of PRP is mainly involved in tannins precipitation Watrelot et al. (2015) observed that the impact of polyphenol concentration on haze formation and precipitation was different for the poly-L-proline (PLP) and BSA. The presence of galloyl groups (Rinaldi et al., 2015) and the kind of interflavanoid bond of proanthocyanidins (de Freitas and Mateus, 2001) are also critical features for the selective ability of tannins to bind different proteins and to be effectively responsible of astringency.

## Conclusion

Results obtained in this study confirmed previous observations on the existence of differences between initial and further oxidation steps and the finding that OCRs are negatively related to the wine content in monomeric and total anthocyanins.

This is the first time that average OCR and, to a lesser extent initial OCR, have been shown to be positively correlated to VRF/mA, T/A ratios and marc pressing.

In addition results obtained in this study confirm a general trend already observed during wine aging and oxidation, about the effect of oxidation on main red wine phenolic classes which follows the trend: monomeric anthocyanins>flavanols and low molecular weight condensed flavanols (VRF)>BSA reactive tannins. The effect on the precipitation of saliva proteins is instead a combination of these activities.

From an enological point of view and in broad practical terms, the stronger the pressing of marc after maceration is, the faster the consumption of oxygen of red wines because of the increase of VRF/mA and T/A ratios. The acceleration of oxidation facilitates the removing of dissolved oxygen from wine with practical implications for further SO_2_ addition and for the management of barrel/bottle aging or micro-oxygenation. Future work should examine the influence of this ratio during aging of wine protected by different level of SO_2_.

## Author contributions

AG planned the study and drafted the manuscript. LP conducted oxidation experiments and carried out polyphenols and acetaldehyde analyses under the supervision of AG and LM. AR performed saliva interaction experiments. LP, AR, and AG analyzed the data. LM supervised the study. All authors read and approved the final manuscript.

### Conflict of interest statement

The authors declare that the research was conducted in the absence of any commercial or financial relationships that could be construed as a potential conflict of interest.

## Footnotes

^1^OIV (2017). Available online at: http://www.oiv.int/en/oiv-life/oiv-2017-reporton-the-world-vitivinicultural-situation

^2^Addinsoft (2009). Available online at: https://www.xlstat.com/fr/
