# Supplementary material for: Evolution of Sangiovese Wines With Varied Tannin and Anthocyanin Ratios During Oxidative Aging
Source: Front Chem. 2018 Mar 15;6:63. doi: 10.3389/fchem.2018.00063 (PMC5862844; doi:10.3389/fchem.2018.00063)
Supplement: Supplementary file 1 [file Table1.docx]

|  | **L*** | | | | **a*** | | | | **b*** | | | | **Chromaticity C*** | | | | **Hue Angle** | | | | **∆E** | | | |
| --- | --- | --- | --- | --- | --- | --- | --- | --- | --- | --- | --- | --- | --- | --- | --- | --- | --- | --- | --- | --- | --- | --- | --- | --- |
| **No Ox** |  |  |  |  |  |  |  |  |  |  |  |  |  |  |  |  |  |  |  |  |  |  |  |  |
| HAf | 66.72 | ± | 1.21 | B* | 43.28 | ± | 0.34 | AB* | 20.92 | ± | 0.26 | B* | 48.07 | ± | 0.22 | AB* | 25.80 | ± | 0.44 | C* |  |  |  |  |
| HAp | 63.07 | ± | 1.69 | B* | 41.05 | ± | 0.58 | B* | 23.43 | ± | 0.94 | B* | 47.28 | ± | 0.14 | B* | 29.72 | ± | 1.32 | BC* |  |  |  |  |
| MAf | 64.44 | ± | 0.11 | B* | 49.00 | ± | 0.15 | A* | 31.58 | ± | 0.15 | A* | 58.30 | ± | 0.20 | A* | 32.80 | ± | 0.06 | A* |  |  |  |  |
| MAp | 80.82 | ± | 0.69 | AB* | 27.33 | ± | 0.96 | A* | 17.16 | ± | 0.62 | C* | 32.27 | ± | 1.14 | AB* | 32.12 | ± | 0.11 | C* |  |  |  |  |
| LAf | 82.40 | ± | 0.13 | A* | 22.50 | ± | 0.18 | B* | 11.54 | ± | 0.10 | C* | 25.29 | ± | 0.19 | C* | 27.15 | ± | 0.17 | BC* |  |  |  |  |
| LAp | 83.82 | ± | 0.09 | AB* | 19.43 | ± | 0.05 | A* | 9.32 | ± | 0.08 | B* | 21.55 | ± | 0.07 | AB* | 25.62 | ± | 0.15 | B* |  |  |  |  |
| **SAT1** |  |  |  |  |  |  |  |  |  |  |  |  |  |  |  |  |  |  |  |  |  |  |  |  |
| HAf | 72.14 | ± | 0.15 | A* | 38.42 | ± | 0.35 | BC | 19.28 | ± | 0.26 | B* | 42.99 | ± | 0.43 | BC* | 26.64 | ± | 0.12 | C* | 7.53 | ± | 0.47 | B |
| HAp | 68.64 | ± | 0.38 | A* | 38.17 | ± | 0.15 | C | 21.69 | ± | 0.12 | D* | 43.90 | ± | 0.18 | C* | 29.61 | ± | 0.08 | C* | 6.62 | ± | 1.02 | BC |
| MAf | 75.95 | ± | 0.92 | A* | 31.66 | ± | 0.31 | B* | 19.27 | ± | 0.17 | B* | 37.06 | ± | 0.35 | B* | 31.32 | ± | 0.03 | B* | 2.02 | ± | 0.65 | A |
| MAp | 80.45 | ± | 0.66 | AB* | 26.02 | ± | 0.07 | AB* | 17.68 | ± | 0.25 | BC* | 31.45 | ± | 0.17 | B* | 34.20 | ± | 0.36 | B* | 1.61 | ± | 0.65 | B |
| LAf | 81.81 | ± | 0.11 | B* | 23.96 | ± | 0.21 | A* | 11.97 | ± | 0.17 | BC* | 26.78 | ± | 0.18 | AB* | 26.54 | ± | 0.43 | C* | 1.64 | ± | 0.11 | AB |
| LAp | 83.83 | ± | 0.84 | B* | 20.05 | ± | 0.91 | A* | 9.68 | ± | 0.37 | AB* | 22.26 | ± | 0.98 | AB* | 25.77 | ± | 0.19 | B* | 1.32 | ± | 0.57 | A |
| **SAT2** |  |  |  |  |  |  |  |  |  |  |  |  |  |  |  |  |  |  |  |  |  |  |  |  |
| HAf | 65.48 | ± | 4.35 | B | 45.45 | ± | 5.49 | A | 26.44 | ± | 4.00 | A | 52.58 | ± | 6.75 | A | 30.10 | ± | 0.88 | A* | 8.99 | ± | 1.34 | AB* |
| HAp | 63.98 | ± | 0.14 | B | 44.17 | ± | 0.32 | A | 27.48 | ± | 0.10 | A | 52.02 | ± | 0.29 | A | 31.88 | ± | 0.19 | A* | 5.50 | ± | 0.21 | C* |
| MAf | 76.36 | ± | 1.02 | A* | 32.20 | ± | 1.10 | B* | 19.52 | ± | 0.29 | B | 37.66 | ± | 1.08 | B* | 31.23 | ± | 0.54 | B* | 2.02 | ± | 0.92 | A |
| MAp | 79.80 | ± | 0.54 | B* | 27.04 | ± | 0.84 | A* | 19.23 | ± | 0.48 | A | 33.18 | ± | 0.96 | A* | 35.43 | ± | 0.17 | A* | 2.34 | ± | 0.24 | AB |
| LAf | 81.36 | ± | 0.36 | B* | 24.12 | ± | 0.69 | A* | 12.37 | ± | 0.59 | AB* | 27.11 | ± | 0.88 | A* | 27.13 | ± | 0.47 | BC* | 2.15 | ± | 0.99 | A |
| LAp | 84.14 | ± | 1.06 | AB* | 20.41 | ± | 1.28 | A* | 9.90 | ± | 0.58 | AB* | 22.69 | ± | 1.41 | AB* | 25.87 | ± | 0.11 | B* | 1.91 | ± | 0.70 | A |
| **SAT3** |  |  |  |  |  |  |  |  |  |  |  |  |  |  |  |  |  |  |  |  |  |  |  |  |
| HAf | 73.68 | ± | 0.07 | A* | 36.12 | ± | 0.15 | C* | 19.97 | ± | 0.07 | B* | 41.27 | ± | 0.15 | C* | 28.94 | ± | 0.11 | B* | 10.07 | ± | 0.70 | A* |
| HAp | 69.67 | ± | 0.35 | A* | 37.07 | ± | 0.26 | D* | 22.40 | ± | 0.25 | CD* | 43.31 | ± | 0.11 | B* | 31.15 | ± | 0.45 | AB* | 8.15 | ± | 0.76 | AB* |
| MAf | 76.88 | ± | 0.52 | A* | 31.71 | ± | 0.48 | B* | 19.28 | ± | 0.21 | B* | 37.12 | ± | 0.32 | B* | 31.30 | ± | 0.64 | B* | 1.32 | ± | 0.15 | A* |
| MAp | 80.88 | ± | 0.22 | AB* | 25.60 | ± | 0.05 | B* | 18.34 | ± | 0.27 | B* | 31.50 | ± | 0.20 | B* | 35.62 | ± | 0.35 | A* | 2.51 | ± | 0.23 | A* |
| LAf | 82.46 | ± | 0.16 | A* | 23.11 | ± | 0.03 | B* | 12.28 | ± | 0.07 | AB* | 26.17 | ± | 0.06 | BC* | 27.99 | ± | 0.12 | B* | 0.99 | ± | 0.24 | B* |
| LAp | 83.73 | ± | 0.15 | AB* | 20.74 | ± | 0.13 | A* | 10.72 | ± | 0.09 | A* | 23.35 | ± | 0.08 | A* | 27.34 | ± | 0.34 | AB* | 1.99 | ± | 0.17 | A* |

Table 1. Main CIELAB values of the Sangiovese wines subjected to four saturation cycles.

Table 3. continued

| **SAT4** |  |  |  |  |  |  |  |  |  |  |  |  |  |  |  |  |  |  |  |  |  |  |  |  |
| --- | --- | --- | --- | --- | --- | --- | --- | --- | --- | --- | --- | --- | --- | --- | --- | --- | --- | --- | --- | --- | --- | --- | --- | --- |
| HAf | 73.37 | ± | 0.47 | A*SS | 36.55 | ± | 0.57 | CS | 20.91 | ± | 0.35 | B* | 42.11 | ± | 0.67 | BC* | 29.78 | ± | 0.04 | AB* | 9.52 | ± | 1.31 | AB |
| HAp | 69.88 | ± | 0.40 | A* | 36.42 | ± | 0.23 | D | 22.80 | ± | 0.31 | BC* | 42.97 | ± | 0.10 | D* | 32.05 | ± | 0.49 | A* | 8.41 | ± | 0.71 | AB |
| MAf | 76.70 | ± | 0.77 | A* | 31.16 | ± | 0.23 | B* | 19.18 | ± | 0.20 | B* | 36.59 | ± | 0.11 | B* | 31.62 | ± | 0.44 | B* | 1.39 | ± | 0.29 | A* |
| MAp | 81.11 | ± | 0.62 | A* | 25.53 | ± | 0.65 | B* | 18.41 | ± | 0.23 | AB* | 31.48 | ± | 0.65 | B* | 35.81 | ± | 0.40 | A* | 2.27 | ± | 0.04 | AB* |
| LAf | 82.32 | ± | 0.19 | A* | 22.99 | ± | 0.05 | B* | 12.78 | ± | 0.28 | A* | 26.31 | ± | 0.11 | AB* | 29.07 | ± | 0.58 | A* | 1.35 | ± | 0.35 | AB |
| LAp | 85.39 | ± | 0.62 | A* | 18.63 | ± | 0.61 | A* | 9.99 | ± | 0.26 | A* | 21.14 | ± | 0.66 | B* | 28.21 | ± | 0.16 | A* | 1.99 | ± | 0.70 | A |

HAf (High Anthocyanins level from free run juice), HAp (High Anthocyanins level from pressed run juice), MAf (Medium Anthocyanins level from free run juice), MAp (Medium Anthocyanins level from pressed run juice), LAf (Low Anthocyanins level from free run juice), LAp (Low Anthocyanins level from pressed run juice). Wines (A, B, C, D) sharing the same letters are not significantly different after each oxygen saturation cycle. *for each saturation cycle, wines (HA, MA and LA) differing for marc pressing (f or p) applied at the end of maceration (p<0.05).
